# Role of Autotaxin in High Glucose-Induced Human ARPE-19 Cells

**DOI:** 10.3390/ijms23169181

**Published:** 2022-08-16

**Authors:** Yang Liu, Reiko Yamagishi, Megumi Honjo, Makoto Kurano, Yutaka Yatomi, Koji Igarashi, Makoto Aihara

**Affiliations:** 1 Department of Ophthalmology, Graduate School of Medicine, The University of Tokyo, Tokyo 113-8655, Japan; 2 Department of Clinical Laboratory Medicine, Graduate School of Medicine, The University of Tokyo, Tokyo 113-8655, Japan; 3 Department of Clinical Laboratory, The University of Tokyo Hospital, Tokyo 113-8655, Japan; 4 Bioscience Division, Reagent Development Department, AIA Research Group, TOSOH Corporation, Ayase 252-1123, Japan

**Keywords:** autotaxin, diabetic retinopathy, human ARPE-19 cells

## Abstract

Autotaxin (ATX) is an enzymatic with lysophospholipase D (lysoPLD) activity. We investigated the role of ATX in high glucose (HG)-induced human retinal pigment epithelial (ARPE-19) cells to explore the pathogenesis of diabetic retinopathy (DR). We performed a quantitative real-time polymerase chain reaction, Western blotting, immunocytochemistry, enzyme-linked immunosorbent assay, cell permeability assay, and transepithelial electrical resistance measurement in HG-induced ARPE-19 cells and compared their results with those of normal glucose and osmotic pressure controls. ATX expression and its lysoPLD activity, barrier function, and expression of vascular endothelial growth factor receptors VEGFR-1 and VEGFR-2 were downregulated, while fibrotic responses, cytoskeletal reorganization, and transforming growth factor-β expression were upregulated, in the HG group. Our results suggest that HG induces intracellular ATX downregulation, barrier dysfunction, and fibrosis, which are involved in early DR and can be targeted for DR treatment.

## 1. Introduction

Diabetic retinopathy (DR) is a complication of diabetes mellitus characterized by pathological changes in the retina, including exudates, swelling, neovascularization, and hemorrhage. In 2020, DR was estimated to affect more than 100 million adults, which is expected to increase to 160 million by 2045 [1]. DR is a leading cause of preventable vision loss and blindness in the working-age population worldwide [2,3,4,5,6,7].

Hyperglycemia is a major risk factor of DR [8,9]. In vitro studies have used the high glucose (HG)-induced human retinal pigment epithelial (RPE) cell line ARPE-19 to investigate the DR pathogenesis [10]. In HG-induced ARPE-19 cells, several pathological changes are associated with DR, including the upregulation of vascular endothelial growth factor (VEGF) [11]; transforming growth factor (TGF) [12,13]; matrix metalloproteinases [14,15,16]; inflammatory cytokines [17]; oxidative stress [18,19,20,21]; and genetic factors [22,23,24]. However, few studies have investigated the relationship between autotaxin (ATX) and DR.

The ATX enzyme is encoded by the *ENPP2* gene, which was first discovered as a motility-stimulating protein in melanoma cells [25]. ATX was later identified based on the lysophospholipase D (lysoPLD) activity, which can catalyze lysophosphatidylcholine (LPC) hydrolysis to produce lysophosphatidic acid (LPA) [26,27]. In general, the ATX–LPA axis has various important physiological and pathological roles in various tissues related to inflammation, migration, fibrosis, or tumorigenesis. ATX is upregulated in the epiretinal membranes and downregulated in the vitreous samples of DR patients [28,29], but its role in the regulation of retinal epithelial cells is unknown.

Recent studies have suggested that several mechanisms are involved in DR pathogenesis, including retinal fibrosis caused by extracellular matrix remodeling [30], increased epithelial-to-mesenchymal transition, the fibrosis of fibrovascular tissue [31], and loss of blood–retinal barrier integrity, which leads to subsequent vascular dysfunction [32]. However, the role of ATX in the aforementioned processes is not clear.

In the present study, we investigated the role of ATX in HG-induced changes in ARPE-19 cells and the related barrier functions, fibrotic response, and remodeling mediators. Our study identified novel effects of ATX regulation and provided insights to support the development of DR treatments.

## 2. Results

### 2.1. Decreased ATX Expression Induced by HG in ARPE-19 Cells

**Figure 1:**
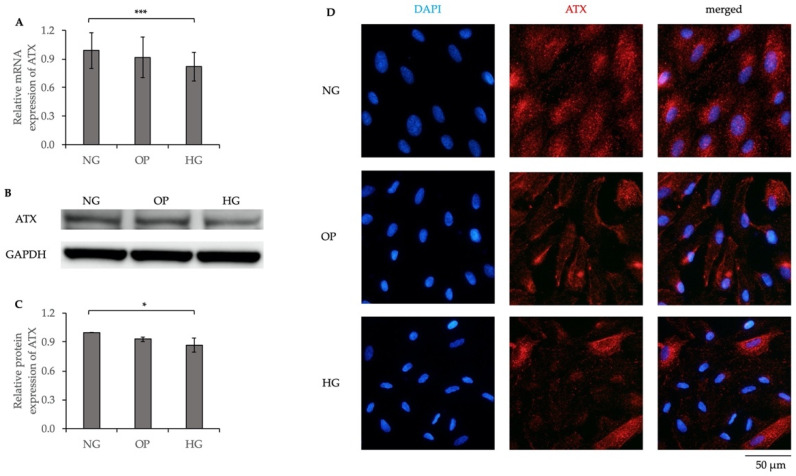
Decreased ATX expression induced by HG in ARPE-19 cells. (**A**) qRT-PCR quantification of ATX mRNA expression relative to that of GAPDH in NG, osmotic pressure (OP) control, and HG groups. (**B**) Representative WB results of ATX protein expression and GAPDH. (**C**) Quantification of WB band intensity of ATX protein expression relative to that of GAPDH. (**D**) ICC results of ATX. The left panels show cells stained with DAPI. The middle panels show cells stained with anti-ATX antibody. The right panels show merged images of anti-ATX and DAPI. Bar, 50 μm. * *p* < 0.05 and *** *p* < 0.001 (ANOVA, followed by Tukey’s multiple comparison test).

A previous study reported a decreased ATX level in proliferative DR (PDR) patients [29]; therefore, we determined the expression level of ATX in HG-induced ARPE-19 cells using a quantitative real-time polymerase chain reaction (qRT-PCR); Western blotting (WB); and immunocytochemistry (ICC). The relative ATX mRNA and protein expression levels were significantly lower in the HG group than the normal glucose (NG) group (*p* < 0.001 and *p* < 0.05, respectively; Figure 1A–C). In addition, ICC showed that ATX expression was significantly downregulated in the HG group (Figure 1D).

### 2.2. Decreased ATX Activity and Increased LPC Level Induced by HG in the Conditioned Medium of ARPE-19 Cells

**Figure 2:**
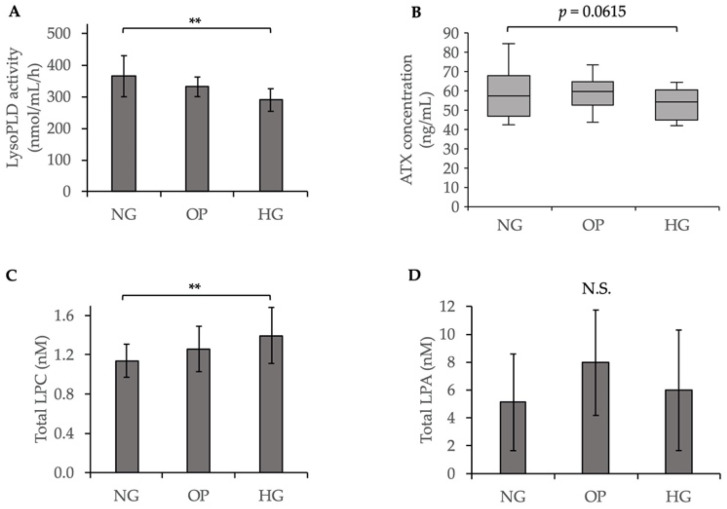
Decreased ATX activity and increased LPC level induced by HG in a conditioned medium of ARPE-19 cells. (**A**) LysoPLD activity (nmol/mL/h) was quantified in a conditioned medium. (**B**) The secreted ATX level in the conditioned medium was determined using AIA system analysis. (**C**) Total LPC concentration in the conditioned medium. (**D**) Total LPA concentration in the conditioned medium. N.S. means statistically not significant. ** *p* < 0.01 (ANOVA followed by Tukey’s multiple comparison test).

Because ATX has lysoPLD activity that transforms LPC to LPA [26,27], we investigated the ATX secretion, lysoPLD activity levels, LPC levels, and downstream LPA levels in a conditioned medium of cultured ARPE-19 cells. The lysoPLD activity was significantly lower in the HG group than the NG group (*p* < 0.01, Figure 2A). The secreted ATX level was also lower in the HG medium than the NG medium, although the difference was not significant (*p* = 0.0615, Figure 2B). The total LPC level was significantly higher in the HG group than the NG group (*p* < 0.01, Figure 2C), while the total LPA level did not differ significantly between the NG and HG groups. LPC 22:6 and LPA 22:6 were the major molecular species detected in the conditioned medium. As shown in Figure S2, the LPC level 22:6 and the LPA level 22:6 did not differ significantly between the NG and HG groups.

### 2.3. Decreased Expression of LPA Receptors (LPARs) Induced by HG in ARPE-19 Cells

**Figure 3:**
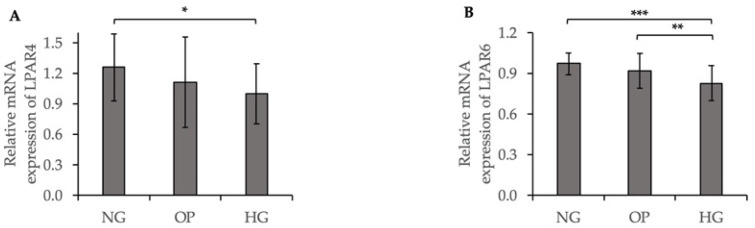
Decreased LPAR expression induced by HG in ARPE-19 cells. qRT-PCR quantification of (**A**) LPAR4 and (**B**) LPAR6 mRNA expression levels relative to that of GAPDH. * *p* < 0.05, ** *p* < 0.01 and *** *p* < 0.001 (ANOVA followed by Tukey’s multiple comparison test).

We further investigated the LPAR expression levels in HG-induced ARPE-19 cells by a qRT-PCR. The relative mRNA expression level of LPAR4 was significantly lower in the HG group than the NG group (*p* < 0.05, Figure 3A). The relative mRNA expression of LPAR6 was significantly lower in the HG group than the NG (*p* < 0.001, Figure 3B) and osmotic pressure (OP) (*p* < 0.01, Figure 3B) groups. As shown in Figure S3, a qRT-PCR quantification of LPAR1, LPAR2, LPAR3, and LPAR5 mRNA expression levels did not differ significantly between the NG and HG groups.

### 2.4. Changes in Cell Permeability and Transepithelial Electrical Resistance (TEER) Induced by HG in ARPE-19 Cells

**Figure 4:**
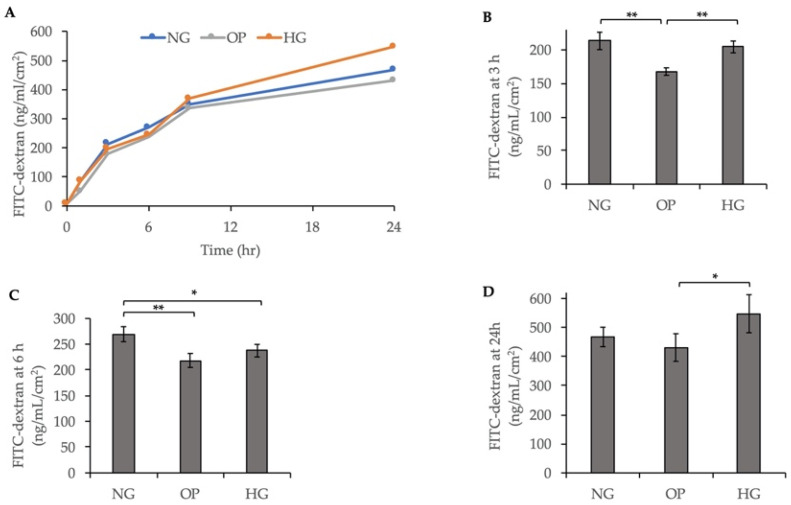
Decreased cell permeability induced by HG in ARPE-19 cells. (**A**) Variation in FITC-dextran concentration at 1, 3, 6, 9 and 24 h after treatment with NG, OP, or HG medium. Cell permeability quantification at (**B**) 3 h, (**C**) 6 h, and (**D**) 24 h post-exposure. * *p* < 0.05 and ** *p* < 0.01 (ANOVA followed by Tukey’s multiple comparison test).

Studies have reported that the upregulation of Rho-Rho kinase induced by the ATX–LPA pathway can lead to the decreased permeability of several cells, including Schlemm’s canal endothelial cells and trabecular meshwork cells [33,34]. We used 4 kDa fluorescein isothiocyanate (FITC)-dextran dye to assess the ARPE-19 cell monolayer permeability. The results showed that HG significantly decreased the concentration of FITC-dextran in the basal side of the ARPE-19 cells at 3 h (*p* < 0.01) and 6 h (*p* < 0.05) compared to the OP and NG groups (Figure 4B,C). At 9 h, the FITC-dextran concentration was higher in the HG group than the NG and OP groups (Figure 4A). At 24 h, HG significantly increased the concentration of FITC-dextran in the basal side of the ARPE-19 cells (*p* < 0.05) compared to the OP group (Figure 4D).

**Figure 5:**
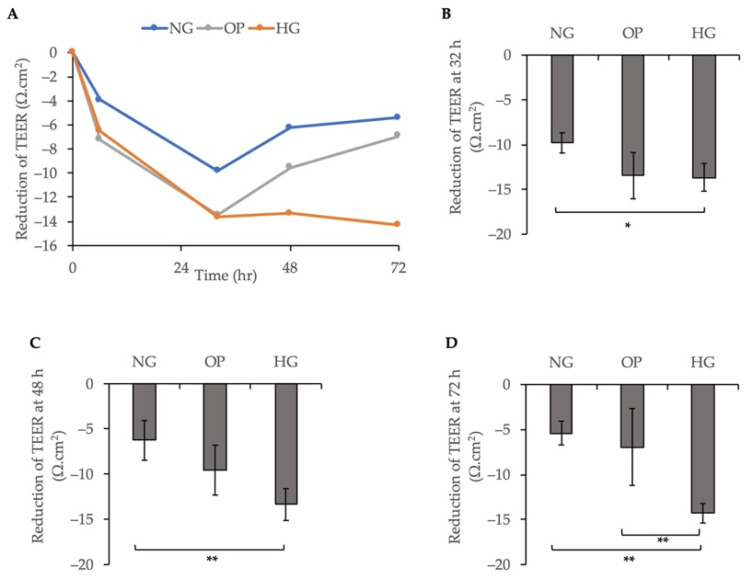
Decreased TEER induced by HG in ARPE-19 cells. (**A**) Variation in TEER at 6, 32, 48, and 72 h after treatment with NG, OP, or HG medium. Quantification of TEER reduction at (**B**) 32 h, (**C**) 48 h, and (**D**) 72 h post-exposure. * *p* < 0.05 and ** *p* < 0.01 (ANOVA followed by Tukey’s multiple comparison test).

TEER was measured at 6, 32, 48, and 72 h to evaluate the barrier function of ARPE-19 cells after treatment with NG, OP, or HG medium. The TEER values decreased significantly within 32 h in all groups (Figure 5A). The TEER values were significantly lower at 32 h (*p* < 0.05); 48 h (*p* < 0.01); and 72 h (*p* < 0.01) in the HG group than the NG group (Figure 5B–D).

### 2.5. Decreased Junction-Associated Protein Expression Induced by HG in ARPE-19 Cells

**Figure 6:**
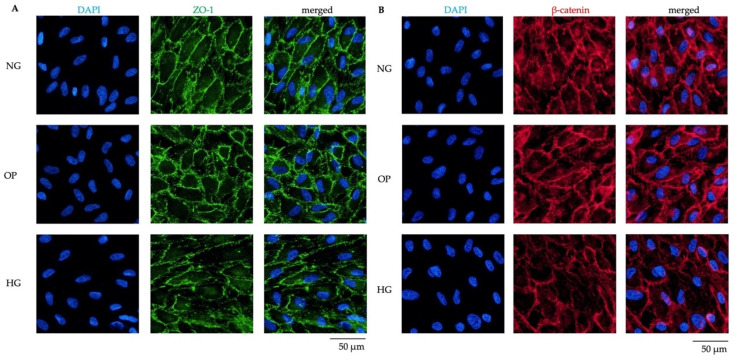
Decreased junction-associated protein expression induced by HG in ARPE-19 cells. (**A**) ICC results of expression of the tight junction protein ZO-1. The left panels show cells stained with DAPI. The middle panels show cells stained with anti-ZO-1 antibody. The right panels show merged images of anti-ZO-1 and DAPI. (**B**) ICC results of the expression of the cell–cell adhesion protein β-catenin. The left panels show the cells stained with DAPI. The middle panels show the cells stained with anti-β-catenin antibody. The right panels show the merged images of anti-β-catenin and DAPI. Bar, 50 μm.

Based on the results of cell permeability and TEER, we examined the effects of HG on tight junctions and cell–cell adhesion-associated protein expression in ARPE-19 cells. ICC revealed the significant downregulation of zonula occludens-1 (ZO-1) (Figure 6A) and β-catenin (Figure 6B) in the HG group compared to the NG and OP groups.

### 2.6. Effects of HG on VEGF Expression in ARPE-19 Cells and Conditioned Medium

**Figure 7:**
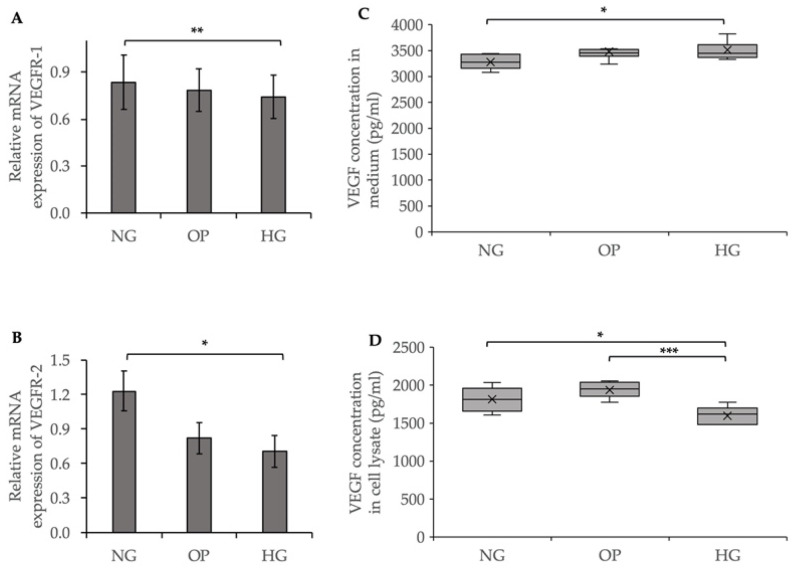
Effects of HG on VEGF expression in ARPE-19 cells and conditioned medium. qRT-PCR quantification of (**A**) VEGFR-1 and (**B**) VEGFR-2 mRNA expression levels relative to that of GAPDH. (**C**) Quantification of VEGF level in the conditioned medium. (**D**) Quantification of VEGF level in cell lysates. * *p* < 0.05, ** *p* < 0.01, and *** *p* < 0.001 (ANOVA followed by Tukey’s multiple comparison test).

To evaluate the effects of HG on VEGF expression, we performed a qRT-PCR of the VEGF receptors (VEGFR-1 and -2) and enzyme-linked immunosorbent assay (ELISA) for VEGF on the conditioned medium and cell lysates. The relative mRNA expression levels of VEGFR-1 (*p* < 0.01) and VEGFR-2 (*p* < 0.05) were significantly decreased in the HG group compared to the NG group (Figure 7A,B). The ELISA results showed that HG treatment significantly increased the VEGF level in the conditioned medium (*p* < 0.05) compared to the NG group, while the VEGF level was significantly reduced in the cell lysates (*p* < 0.05) in the HG group (Figure 7C,D).

### 2.7. Increased Fibrotic Responses and Cytoskeletal Reorganization Induced by HG in ARPE-19 Cells

**Figure 8:**
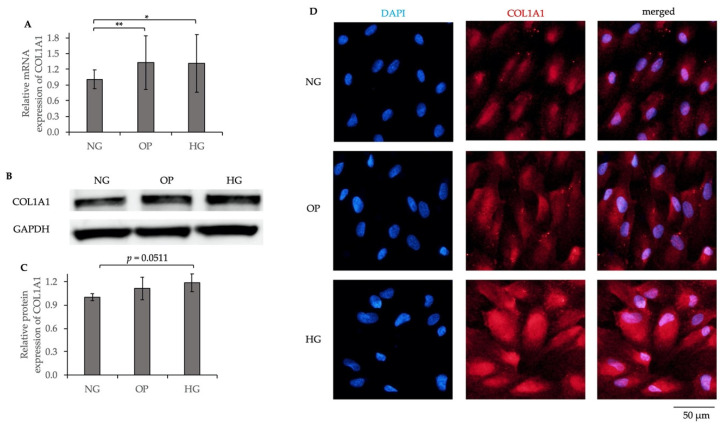
Increased COL1A1 expression was induced by HG in ARPE-19 cells. (**A**) qRT-PCR quantification of COL1A1 mRNA expression relative to that of GAPDH. (**B**) Representative WB results of COL1A1 protein expression and GAPDH. (**C**) Quantification of WB band intensity of COL1A1 protein expression relative to that of GAPDH. (**D**) ICC results of COL1A1. The left panels show the cells stained with DAPI. The middle panels show the cells stained with the anti-COL1A1 antibody. The right panels show merged images of anti-COL1A1 and DAPI. Bar, 50 μm. * *p* < 0.05 and ** *p* < 0.01 (ANOVA followed by Tukey’s multiple comparison test).

We investigated whether HG induces fibrotic responses or cytoskeletal changes in ARPE-19 cells using a qRT-PCR, WB, and ICC. The relative mRNA expression of collagen type I alpha I chain (COL1A1) was significantly higher in the HG group compared to the NG group (*p* < 0.05, Figure 8A). The relative protein expression of COL1A1 was higher in the HG group compared to the NG group, although the difference was not significant (*p* = 0.0511, Figure 8B,C). In addition, ICC revealed upregulated COL1A1 expression in the HG group (Figure 8D).

**Figure 9:**
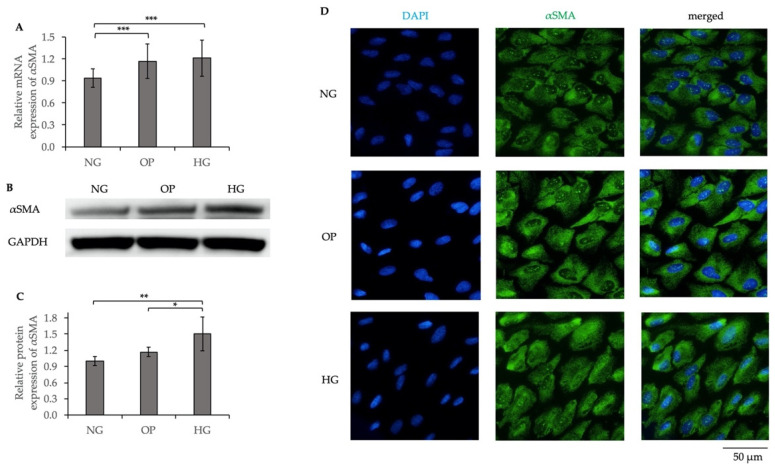
Increased αSMA expression induced by HG in ARPE-19 cells. (**A**) qRT-PCR quantification of αSMA mRNA expression relative to that of GAPDH. (**B**) Representative WB results of αSMA protein expression and GAPDH. (**C**) Quantification of WB band intensity of αSMA protein expression relative to that of GAPDH. (**D**) ICC results of αSMA. The left panels show cells stained with DAPI. The middle panels show cells stained with anti-αSMA antibody. The right panels show merged images of anti-αSMA and DAPI. Bar, 50 μm. * *p* < 0.05, ** *p* < 0.01 and *** *p* < 0.001 (ANOVA followed by Tukey’s multiple comparison test).

The relative mRNA and protein expression levels of alpha-smooth muscle actin (αSMA) were significantly upregulated (*p* < 0.001 and *p* < 0.01, respectively) in the HG group compared to the NG group (mRNA) and NG and OP groups (protein), respectively (Figure 9A–C). ICC revealed upregulated αSMA expression in the HG group compared to the NG group (Figure 9D).

**Figure 10:**
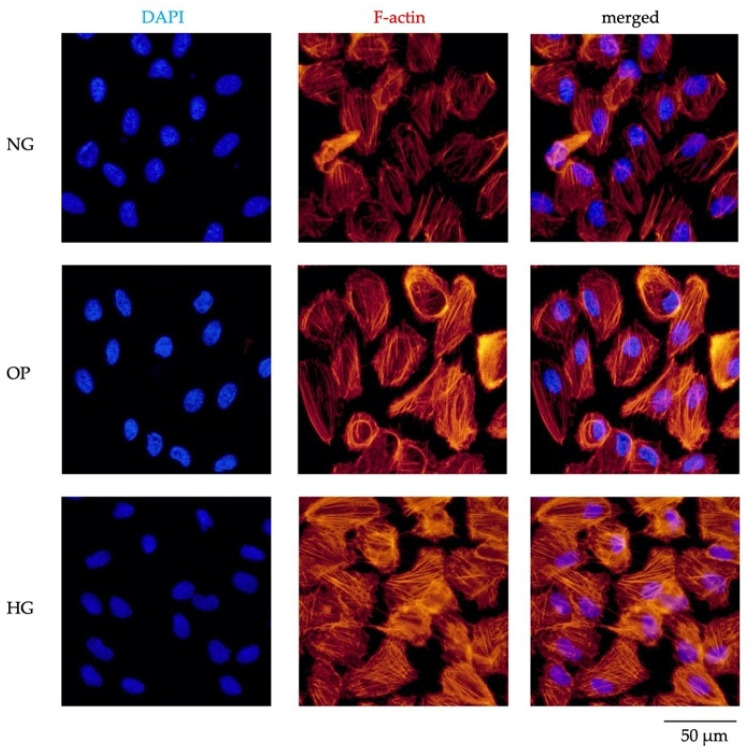
ICC results of F-actin. The left panels show cells stained with DAPI. The middle panels show cells stained with anti-F-actin antibody. The right panels show merged images of F-actin and DAPI. Bar, 50 μm.

ICC also revealed phalloidin-labeled actin filaments in anti-F-actin, indicating that HG induced cytoskeletal reorganization (Figure 10).

### 2.8. Increased TGF-β Expression Induced by HG in ARPE-19 Cells

**Figure 11:**
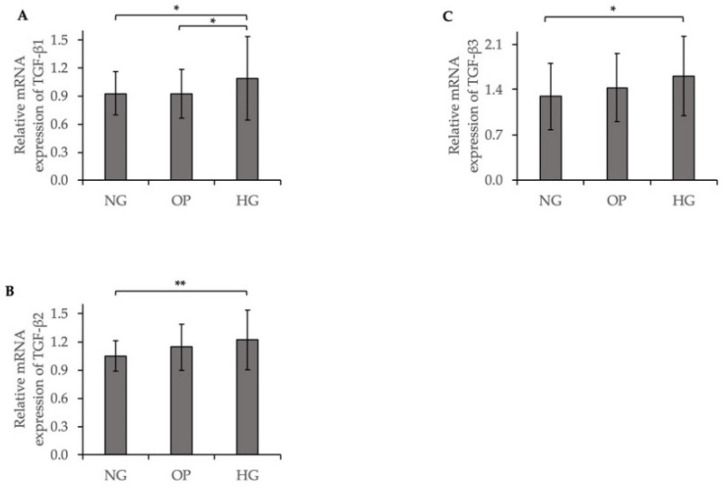
Increased TGF-β expression induced by HG in ARPE-19 cells. qRT-PCR quantification of (**A**) TGF-β1, (**B**) TGF-β2, and (**C**) TGF-β3 mRNA expression levels relative to that of GAPDH (ANOVA followed by Tukey’s multiple comparison test). * *p* < 0.05 and ** *p* < 0.01 (ANOVA followed by Tukey’s multiple comparison test).

To clarify the potential mechanism of increased fibrotic responses induced by HG, we investigated TGF-β expression using a qRT-PCR. The relative mRNA expression levels of TGF-β1 (*p* < 0.05); TGF-β2 (*p* < 0.01); and TGF-β3 (*p* < 0.05) were significantly increased in the HG group compared to the NG group (Figure 11).

## 3. Discussion

ATX is a secreted protein that has lysoPLD enzymatic activity [26,27]. Studies have reported that ATX is important in the regulation of glucose homeostasis in adipocytes [35,36,37,38]. The ATX–LPA pathway mediates cell migration, proliferation, and apoptosis, as well as inflammation, angiogenesis, and fibrosis. We recently reported the specific role of ATX and its trans-signaling with TGF-β in glaucoma subtypes with elevated intraocular pressure by regulating the fibrotic response in trabecular meshwork cells [33,39,40,41]. Only a few studies have investigated the relationship between ATX and DR pathogenesis. El-Asrar et al. reported increased ATX level in the vitreous and epiretinal membrane samples of PDR patients. The ATX–LPA signaling pathway may be an important biomarker of DR development and progression [28,29]. However, the precise role of ATX in the DR pathogenesis is not clear.

In the present study, we found that HG induced the downregulation of ATX expression and lysoPLD activity, and the upregulation of total LPC level in ARPE-19 cells (Figure 1 and Figure 2), while total LPA level did not differ significantly compared with the NG group. A cell viability assay showed no significant differences among the three groups (Figure S1), suggesting that HG did not decrease the number of cultured ARPE-19 cells; however, the expression level of ATX was downregulated in HG-induced ARPE-19 cells. These results are in line with those of previous studies that showed that the ATX level was significantly lower in the vitreous samples of patients with PDR with active neovascularization and inactive PDR, compared to that in nondiabetic patients; however, the levels of acylglycerol kinase and VEGF were increased in patients with PDR and active neovascularization [29]. Since ATX has lysoPLD activity to convert LPC into LPA, increased LPC levels in the conditioned medium of the HG group suggested the overaccumulation of LPC in the extracellular region, in accordance with the downregulation of ATX expression and its lysoPLD activity. However, as shown in Figure S2, the LPC level 22:6 and the LPA level 22:6 did not differ significantly between the HG and NG groups. It may be attributable to the endogenous production related with PA-PLA1α [42] or the acylglycerol kinase pathway [29]. In addition, the basal LPC levels in the medium might have been low to contribute to LPA production via the alteration of ATX activity. Further studies will be needed, including in vivo experiments to clarify this issue. We further investigated LPAR expression and found that LPAR4 and LPAR6 were significantly downregulated in the HG group compared to the NG group (Figure 3), whereas LPAR1, LPAR2, LPAR3, and LPAR5 levels were not significantly different between the HG and NG groups (Figure S3). These results are consistent with those of a study showing the downregulation of the ATX–LPA pathway with HG treatment [43].

We also investigated the effects of HG treatment on cell permeability using FITC-dextran flux assay and TEER. The FITC-dextran flux assay and TEER values demonstrated that HG induced a significant decrease in permeability within 6 h (Figure 4) and 72 h (Figure 5), respectively, compared to the NG group. ICC for ZO-1 and β-catenin further confirmed the change in barrier function in HG-treated ARPE-19 cells (Figure 6). As shown in Figure 4A, the FITC-dextran level started to increase at 9 h and was higher in the HG group than the NG and OP groups. At 24 h, the FITC-dextran concentration in the HG group was significantly higher than the OP group (Figure 4D). Therefore, the change in cell permeability was detected by FITC-dextran flux later compared to TEER measurement. In addition, the TEER values of the three groups began to increase after 72 h and remained elevated until 10 days. Notably, the levels were higher in the HG group than the NG group after day 7 (Figure S4). The long-term effects of HG on the barrier function of ARPE-19 cells and reversal thereof are consistent with previous studies with longer observation periods [44,45,46]. However, most previous studies measured TEER values after switching to the HG medium for at least 1 week. Thus, our study was the first to analyze the early effects (within 1 week) of HG on ARPE-19 cells using TEER. Based on our recent study of the effects of ATX on the barrier function in monkey Schlemm’s canal endothelial cells and human trabecular meshwork cells [33,34,40], we hypothesized that the early decrease in TEER in HG-induced ARPE-19 cells could be related to ATX downregulation. Additional research is required to confirm this relationship.

Because of the role of VEGF in the DR pathogenesis [47] and the interactive effects of VEGF and ATX on the regulation of cellular responses [48], we evaluated the effects of HG on VEGF expression in ARPE-19 cells. As shown in Figure 7A,B, VEGFR-1 and VEGFR-2 expression levels were significantly decreased in the HG group, while VEGF-A and VEGF-B expression levels were not changed (Figure S5). An ELISA of medium and cell lysates showed an increased and decreased accumulation of secreted VEGF in the extracellular and intracellular regions, respectively (Figure 7C,D), suggesting that the VEGF levels were similar in the conditioned medium and DR patients. A previous study showed that the VEGF and LPA levels were significantly higher in the vitreous samples of PDR patients compared to those in control patients without diabetes, in line with the present results [29]. Our finding of increased LPA level in DR patients was not in line with the decrease in LPA and LPAR levels by ATX downregulation. In the present study, we evaluated the short-term effects of HG (i.e., within 48 h) and found downregulated ATX and LPAR functions. It is possible that an LPA-generating enzyme acylglycerol kinase, which is elevated in DR patients, may have caused an increase in the LPA level induced by long-term HG in DR patients [29]. The downregulation of ATX results in decreased VEGFR-1 and VEGFR-2 expression levels in ovarian cancer cells [43]. A feedback loop exists between VEGF and the ATX–LPA pathway. LPA induces VEGF by activating hypoxia inducible factor-1 [49]. In addition, VEGF regulates ATX transcription and secretion via VEGFR-2 [43,48]. Further studies are needed to elucidate the exact relationship between these signaling pathways.

We compared the fibrotic responses and cytoskeletal changes in NG-, OP-, and HG-treated ARPE-19 cells. The COL1A1 and αSMA mRNA and protein expression levels were significantly upregulated by HG. ICC showed significantly increased expression levels of COL1A1 (Figure 8D); αSMA (Figure 9D); and F-actin (Figure 10) in the HG group. Our results suggest that fibrogenic activity and cytoskeletal reorganization are increased in PDR, which is indicative of pathological angiogenesis [50,51,52,53,54,55,56]. In addition, HG induced significant upregulations of TGF-β1, TGF-β2, and TGF-β3 levels in ARPE-19 cells (Figure 11), whereas the TGF-βi level did not differ significantly among the three groups (Figure S6). The elevated TGF-β levels in DR may be related to fibrosis [57] and barrier dysfunction [58]. We recently reported trans-regulation between ATX and the TGF-β signaling pathway in trabecular meshwork cells. Further studies are needed to determine the role of signaling feedback in DR.

Our study had several limitations. First, most experiments were conducted after treatment with the conditioned medium for 48 h, except for the cell permeability assay and TEER measurement. Prolonged observations are needed to determine the effects of HG. Second, we did not perform additional experiments to investigate the roles of sphingosine 1-phosphate [59] and other angiogenic factors (such as basic fibroblast growth factor) [60]. Additional studies are needed to determine the roles of these factors in HG-induced ARPE-19 cells. Third, we performed experiments only on ARPE-19 cells. Future studies should include a combination of in vitro and in vivo experiments, including those on DR animal models. Fourth, our data only suggested the positive correlation between ATX expression and TEER values after 24 h. The underlying mechanism still remains unclear and future research is necessary for interpretation. Last, we could not elucidate the ATX downregulation directly with alterations of fibrotic changes, cytoskeleton reorganization, VEGF, and TGF expression. We will continue investigation if novel reagents targeting ATX activation are developed.

In summary, our study is the first to demonstrate the role of ATX in HG-induced ARPE-19 cells and the short-term effects of HG on barrier function. Our study showed downregulated VEGFR-1 and VEGFR-2 expression levels in ARPE-19 cells, suggesting that VEGF regulation is associated with ATX expression. HG-induced ARPE-19 cells showed an upregulation of COL1A1, αSMA, F-actin, and TGF-β levels, suggesting that fibrogenic changes and TGF-β are related to the DR pathogenesis and may be targeted for DR treatment.

## 4. Materials and Methods

### 4.1. Cell Culture and Treatment

The human RPE cell line ARPE-19 was obtained from American Type Culture Collection (Manassas, VA, USA). The ARPE-19 cells were cultured in Dulbecco′s Modified Eagle′s Medium (Sigma-Aldrich, St. Louis, MO, USA) containing 10% fetal bovine serum (Biowest, Nuaillé, France) and 1% antibiotic antimycotic solution (ThermoFisher Scientific, Waltham, MA, USA) in a humidified 37 °C incubator with 5% CO_2_. The medium was changed every 2 days. We used cells between passages 16 and 24 for the experiments.

The ARPE-19 cells were maintained until 70–80% confluence and then exposed to serum-free starvation for 24 h, followed by treatment with NG (5.5 mM of D-glucose); OP control (5.5 mM of D-glucose plus 19.5 mM of mannitol); or HG (25 mM of D-glucose) for 48 h, unless stated otherwise. The experiments were conducted at least three times to confirm the consistency of the results.

### 4.2. qRT-PCR

Total RNA was extracted from the cells using ISOGEN (Nippon Gene Co. Ltd., Tokyo, Japan), according to the manufacturer’s protocol. The cDNA was synthesized from isolated mRNA using ReverTra Ace qPCR RT Master Mix and gDNA remover (TOYOBO, Osaka, Japan). mRNA expression was quantified using a qRT-PCR of cDNA with TB Green Premix Ex Taq II and the Thermal Cycler Dice Real-Time System II (TaKaRa Bio, Kusatsu, Japan) with the ΔΔCt method. The primers were purchased from Hokkaido System Science (Hokkaido, Japan). The primer sequences are shown in Table S1. Note that LPAR primer sequences were the same as those previously described [42,61]. The data were normalized to the glyceraldehyde-3-phosphate dehydrogenase (GAPDH) expression level.

### 4.3. WB

Cell lysates were collected using a radioimmunoprecipitation assay buffer (ThermoFisher Scientific) containing complete protease inhibitor cocktail (Roche Diagnostics, Mannheim, Germany). The lysates were sonicated and centrifuged at 4 °C and 12,000 rpm for 20 min. The protein levels in the supernatant were measured using the Pierce BCA Protein Assay Kit (ThermoFisher Scientific).

Equal quantities of protein samples were modulated using 2-mercaptoethanol and NuPAGE LDS Sample Buffer, heated at 85 °C, and loaded on NuPAGE 4 to 12% Bis-Tris Gel (Invitrogen, Carlsbad, CA, USA). Sodium dodecyl sulfate–polyacrylamide gel electrophoresis was performed according to the manufacturer’s instructions. The protein bands were transferred to polyvinylidene difluoride membranes (Cytiva, Buckinghamshire, UK). The membranes were blocked and incubated with the primary antibody overnight at 4 °C. The primary antibodies were anti-ATX (1:500, Clone4F1; MBL, Nagoya, Japan; Clone1F8; Abcam, Cambridge, MA, USA); anti-COL1A1 (1:1000; Rockland Immunochemicals, Limerick, PA, USA); and anti-αSMA (1:1000; Dako, Carpinteria, CA, USA and Sigma-Aldrich).

After washing with TBST, the membranes were incubated with the corresponding horseradish peroxidase (HRP)-conjugated secondary antibody (1:2000; ThermoFisher Scientific) and exposed to the ECL substrate (ThermoFisher Scientific). The protein bands were detected using the Image Quant LAS 4000 mini (GE Healthcare, Chicago, IL, USA). The membranes were stripped of the antibodies using the WB Stripping solution (Nacalai tesque, Kyoto, Japan) and incubated with the rabbit monoclonal antibody GAPDH (1:1000, Cell Signaling Technology, Danvers, MA, USA), followed by the HRP-conjugated goat anti-rabbit IgG (H+L) secondary antibody (1:5000; Invitrogen, Rockford, IL, USA). The band intensities were quantified using ImageJ 2.0.0 (National Institutes of Health, Bethesda, MD, USA), and the results were expressed relative to the loading control GAPDH.

### 4.4. ICC

The cells were cultured in 24-well plates with a 13-mm cover glass inside. After treatment with NG, OP, or HG medium for 48 h, the cells were fixed in ice cold 4% paraformaldehyde for 15 min, permeabilized with 0.3% Triton X-100 for 15 min, and blocked in Blocking One Histo (Nacalai tesque) for 30 min. The primary antibodies were anti-ATX (1:1000, Clone1F8; Abcam); anti-COL1A1 (1:200; Rockland Immunochemicals); anti-α-SMA (1:500; Dako); anti-rhodamine phalloidin (7:1000; Cytoskeleton, Inc., Denver, CO, USA); anti-ZO-1 (1:1000; Proteintech, Rosemont, IL, USA); anti-β-catenin (1:200; Merck Millipore, Temecula, CA, USA); and anti-E-cadherin (1:200; Bioss, Woburn, MA, USA). The secondary antibodies were Alexa Fluor 488 and 594 (1:1000; ThermoFisher Scientific). The nuclei were stained with 4′,6-diamidino-2′-phenylindole dihydrochloride (DAPI; 1 μg/mL; Dojindo, Rockville, MD, USA). The immunostained slides were observed and images were obtained using a BZ-9000 fluorescence microscope (Keyence, Osaka, Japan).

### 4.5. Cell Viability Assay

The viability of the cells prepared in 96-well plates was determined using the Cell Counting Kit-8 (Dojindo, Kumamoto, Japan). After incubation for 3 h at 37 °C, the data were collected at 450 nm using a multimode microplate reader (2030 ARVO X3; Perkin Elmer, Kanagawa, Japan).

### 4.6. ELISA

VEGF expression was measured using the Human VEGF Quantikine ELISA Kit (R&D Systems, Minneapolis, MN, USA) in accordance with the manufacturer’s instructions. The concentration in each sample was analyzed using a multimode microplate reader (2030 ARVO X3; Perkin Elmer) at 450 nm with a wavelength correction of 570 nm. The results were calculated by constructing a standard curve.

### 4.7. AIA System Analysis and LC-MS/MS Analysis

The ATX levels in the conditioned medium and cell lysates of cultured ARPE-19 cells were determined using a two-site immunoenzymatic assay, a sensitive anti-ATX monoclonal antibody, and a Tosoh AIA system automated analyzer (Tosoh, Tokyo, Japan), as described previously [39,62,63,64]. The quantification of LPC and LPA levels in the conditioned medium was performed as previously described in detail [63,65,66] using an LC-MS/MS analysis. We identified each LPC and LPA species and monitored 12 acyl chains: 14:0, 16:0, 16:1, 18:0, 18:1, 18:2, 18:3, 20:3, 20:4, 20:5, 22:5, and 22:6. We calculated the concentrations of LPA according to the area ratio relative to 10 μM 17:0 LPC and 1 μM 17:0 LPA (Avanti, Alabaster, AL, USA) as an internal standard.

### 4.8. Measurement of lysoPLD Activity

The ATX activity in the cultured medium was determined based on the lysoPLD activity, as previously described [26,67]. Briefly, the lysoPLD activity was assessed by measuring choline liberation from the substrate LPC. The reactions were performed using 50-μL aliquots; 10 μL of sample was incubated with 2 mM of 1-myristoyl (14:0) LPC (Avanti, Alabaster, AL, USA) in the presence of 100 mM of Tris-HCl (pH 9.0) for 3 h at 37 °C. The liberated choline was detected using an enzymatic photometric method with choline oxidase (Asahi Kasei, Tokyo, Japan); horseradish peroxidase (TOYOBO); 4-aminotipyrine (FUJIFILM Wako, Osaka, Japan); and TOOS reagent (Dojindo) as a hydrogen donor. The choline concentration was estimated using absorption spectrometry.

### 4.9. Measurement of Monolayer Cell Permeability and TEER

The cells were grown on a 12-mm Transwell with 0.4-μm pore polyester membrane inserts (Corning, Kennebunk, ME, USA) until confluent. After starvation, the cell monolayers were exposed to the conditioned medium. A 4 kDa FITC-dextran dye (50 μM; Sigma-Aldrich) was applied to the apical compartment of the wells. The medium was collected from the basal side for fluorescence measurements at 1, 3, and 6 h after adding the dye, and the same volume of fresh conditioned medium was replaced. The FITC-dextran concentration in the collected medium was measured using a multimode microplate reader (2030 ARVO X3; Perkin Elmer) at an excitation wavelength of 485 nm and an emission wavelength of 535 nm. The TEER in each well was measured at 6, 32, 48, and 72 h using a Millicell ERS-2 volt-ohm meter (Merck Millipore).

### 4.10. Statistical Analysis

Statistical analyses were performed using JMP Pro 16 software (SAS Institute, Cary, NC, USA). The experimental data are presented as the mean ± standard deviation, unless stated otherwise. Differences in the data among the groups were analyzed using an analysis of variance (ANOVA) and Tukey’s post hoc test. *p* < 0.05 was considered statistically significant.

## Supplementary Materials

The following supporting information can be downloaded at: https://www.mdpi.com/article/10.3390/ijms23169181/s1.
