# Supplementary material for: Role of Autotaxin in High Glucose-Induced Human ARPE-19 Cells
Source: Int J Mol Sci. 2022 Aug 16;23(16):9181. doi: 10.3390/ijms23169181 (PMC9409272; doi:10.3390/ijms23169181)
Supplement: Supplementary file 1 [file ijms-23-09181-s001.zip › ijms-1856825-supplementary.pdf]

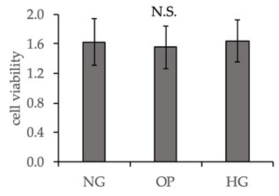

Figure S1. Cell viability assay results were not significantly different among the NG, OP, and HG groups. N.S. means statistically not significant.

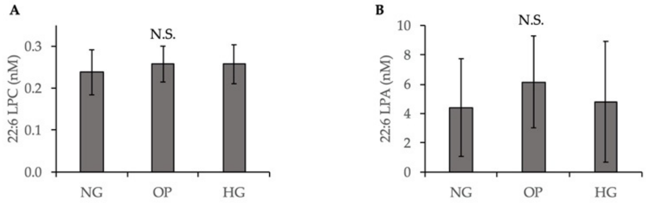

Figure S2. The LPC level 22:6 (A) and the LPA level 22:6 (B) did not differ significantly between the NG and HG groups.

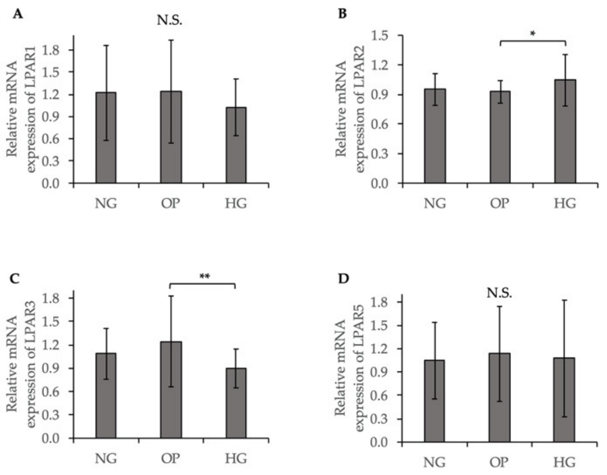

Figure S3. LPAR expression induced by HG in ARPE-19 cells. qRT-PCR quantification of (A) LPAR1, (B) LPAR2, (C) LPAR3, and (D) LPAR5 mRNA expression levels relative to that of GAPDH. * $p < 0.05$ and ** $p < 0.01$ (ANOVA followed by Tukey's multiple comparison test).

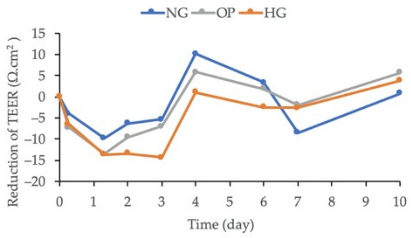

Figure S4. Changes in TEER value at 6, 32, 48, and 72 h and at 4, 6, 7, and 10 days after treatment with NG, OP, and HG media.

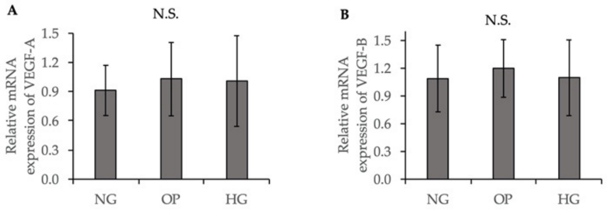

Figure S5. Effects of HG on VEGF expression in ARPE-19 cells. (A) qRT-PCR quantification of (A) VEGF-A and (B) VEGF-B mRNA expression levels relative to that of GAPDH.

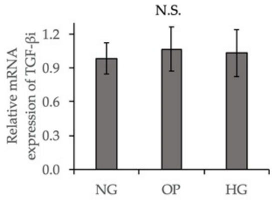

Figure S6. TGF-βi expression induced by HG in ARPE-19 cells. qRT-PCR quantification of TGF-βi mRNA expression relative to that of GAPDH.

Table S1. Primer sequences used for qRT-PCR.

Primers	Forward 5′-3′	Reverse 5′-3′
GAPDH	AATTCCATGGCACCGTCAAG	ATCGCCCCACTTGATTTTGG
ATX	ACAACGAGGAGAGCTGCAAT	AGAAGTCCAGGCTGGTGAGA
COL1A1	CAGCCGCTTCACCTACAGC	TTTTGTATTCAATCACTGTCTTGCC
α-SMA	TTGTCCACCGCAAATGCTTC	AGGTAACGAGTCAGAGCTTTGG
VEGF-A	TTCTGCTGTCTTGGGTGCAT	ATTCTGCCCTCCTCCTTCTG
VEGF-B	CAGTGCTGTGAAGCCAGACA	GGAGTGGGATGGGTGATGT
VEGFR-1	ACTCCCACACTAGGCACAC	TGAGAAGTAGGCATCTGTACTG
VEGFR-2	ACAGCCTTTTTGTTGCAGTGC	TTCAGGCACCTATGCCTGCAC
TGF-β1	CCCAGCATCTGCAAAGCTC	GTCAATGTACAGCTGCCGCA
TGF-β2	TGCCGCCCTTCTTCCCCTC	GGAGCACAAGCTGCCCACTGA
TGF-β3	GGTTTTCCGCTTCAATGTGT	TATAGCGCTGTTTGGCAATG
TGF-βi	GTCCACAGCCATTGACCTTT	GAGTTTCCAGGGTCTGTCCA
